# Bilateral Stress Fractures of the Tibia in a Long-Distance Runner

**DOI:** 10.7759/cureus.70599

**Published:** 2024-10-01

**Authors:** Abdullah Altuwairqi

**Affiliations:** 1 Orthopedic Surgery, King Abdulaziz University Faculty of Medicine, Jeddah, SAU

**Keywords:** case report, intramedullary nailing, long-distance running, stress fracture, tibia

## Abstract

Posterior tibial stress fractures, which are less common than anterior tibial stress fractures, generally have a favorable outcome for resuming athletic activities. Complete fractures are uncommon. A male athlete, age 21, who competes in long-distance running for college athletics, reported experiencing discomfort during training three weeks prior. He covered up to 300 km a week in a balanced manner on the streets and mountains. Even though he had posterior tibial stress fractures, he continued to run, ignoring the pain in his lower legs. After suffering a right lower leg injury during a session of long-distance running, the athlete was sent to the emergency room a year later. He experienced bilateral tibial stress fractures, one of which was fully developed and the other incomplete, at the same time. This is a case of a rather uncommon situation involving bilateral posterior stress fractures managed surgically by exchange intramedullary nailing. One side had a full fracture while the other had a partial fracture. After surgical procedures, the patient was able to start lightly running three months later and was symptom-free five months later. After a year, he could begin running long-distance again. Compared to anterior tibial stress fractures, posterior tibial cortical fractures seem more prevalent among runners and adapt better to conservative therapy. We suggest that if individuals continue receiving excessive training, close, careful monitoring is required.

## Introduction

Tibia stress fractures are microscopic fractures that do not entirely heal when a bone is subjected to repeated strain [1]. Due to cyclical load and insufficient rest, these medical conditions are frequently observed in athletes and new military personnel. Although the precise mechanism is still unknown, during extensive exercise, bone resorption surpasses bone production [2]. Usually, the patient begins complaining of mild pain that worsens when they exercise harder. With repeated injuries over time, the athlete will eventually experience chronic discomfort in the injured area that does not go away with rest. The diaphyseal region is thought to be low-risk, where tibia stress fractures usually occur [3].

Stress fractures are a common incidence in highly dedicated athletes. Long-distance running is an increasingly common form of athletic activity and recreation in the general population [4]. Because of its accessibility and the increased interest in disease prevention, it has become progressively more common. Long-distance running has been linked to many health advantages, but it can also result in injuries [5].

Depending on where they occur, tibial stress fractures occur in two categories: anterior and posterior, resulting in anterior and posterior/posteromedial stress fractures [6]. Athletics involving repeated jumping are more likely to suffer anterior stress fractures, which are marked by a protracted healing process brought on by excessive fiber growth. Located on the anterior, stress side of the tibial shaft, anterior cortical fractures are not as prevalent as posterior stress fractures. They are susceptible to delayed union and nonunion, and they frequently heal poorly because of the constant tension imposed by relatively weak vascular and posterior muscular forces. Anterior tibial stress fractures may occasionally develop into full fractures [7].

This case study focuses on a long-distance runner athlete who suffered a bilateral stress fracture. One side of the fracture was complete while the other was incomplete. The athlete underwent surgical treatment with exchange intramedullary nailing to fix the fracture.

## Case presentation

**Figure 1:**
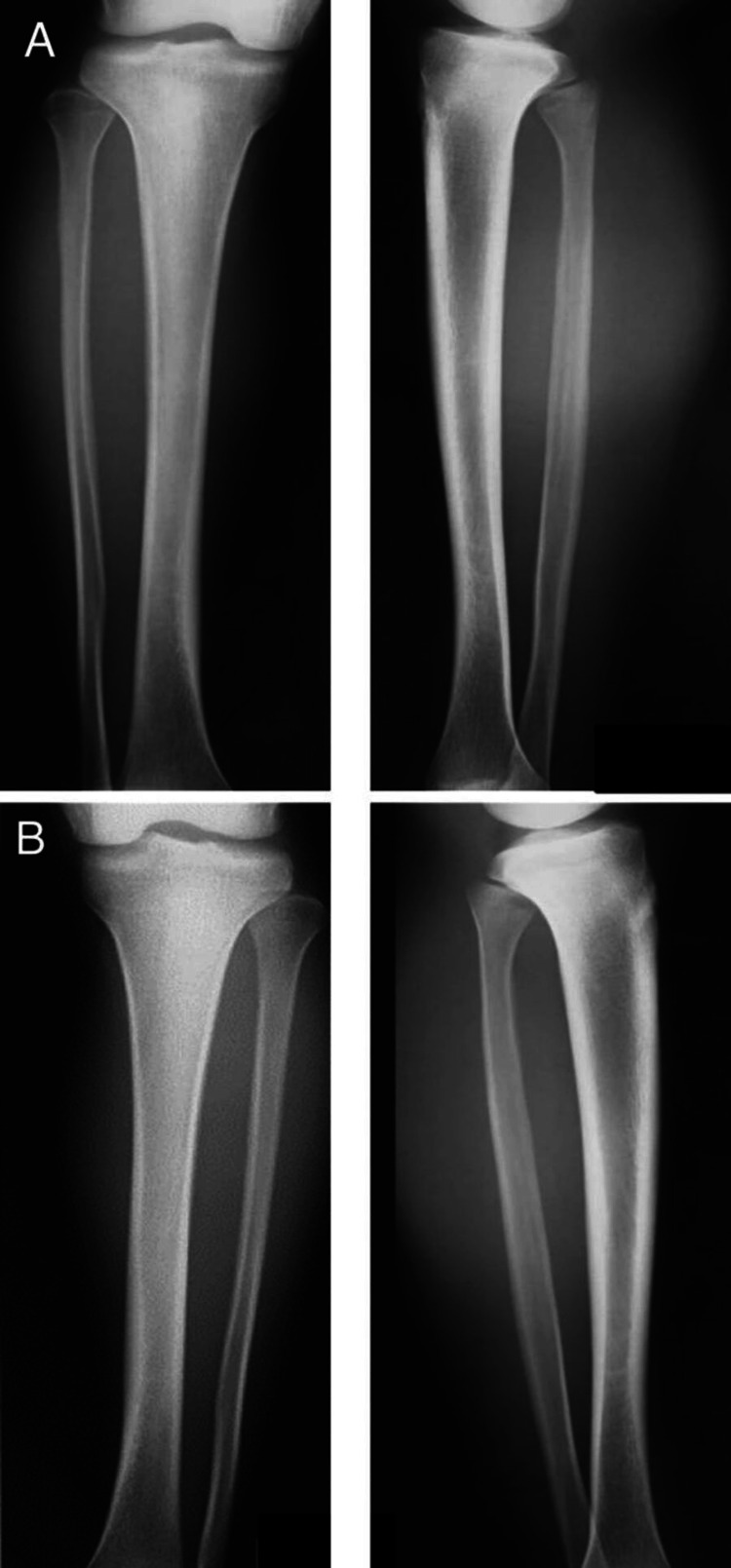
Radiographs of suspected tibial stress fracture no clear abnormality of the tibia in the anteroposterior and lateral views (left): Anteroposterior; (right): Lateral

A male college athlete, age 21, who competes in long-distance running, reported experiencing pain after three weeks of training. He was running cross-country and on trial tracks at a maximum speed of 300 km/week in a uniform manner. He had taken analgesics, but his lower leg pain did not go away during exercise, so he came to our hospital for emergency treatment (Figure 1). The tibia's radiographs did not show any obvious abnormalities; however, short tau inversion recovery (STIR) magnetic resonance imaging (MRI) identified a high-intensity region in the distal part of the tibia, leading to the confirmation of shin splints and stress fractures. The individual was told to stop training while outpatient follow-up care was provided for the injury in a cautious manner. Subsequent radiographs were examined after two and three months. Following three months, he decided to resume running because the fracture healed following this treatment without any problems. He was once more told to discontinue training when imaging at six months revealed enlargement of the bone cortex in the posterior part of the distal portion of the left tibia and the posterior of the right tibia. But after six months, he stopped going to the outpatient facility by himself.

One year after the first assessment, he was evaluated in the emergency room after suffering an injury to his right lower leg during a middle-distance race. He said that just as the race was getting started when he had turned the first curve, he felt a "snap" in his right calf, which made it impossible for him to run any longer. He collapsed and had to give up. He stated that his right leg was twisted unnaturally. His lower limb malformations made it hard for him to run, thus our emergency department admitted him. Upon admission to the hospital for treatment, an X-ray examination revealed a proximal one-third of the left leg with a substantially displaced horizontal fracture; thankfully, there was no bleeding wound. The patient had not felt any kind of discomfort or pain in his left lower leg when exercising before the day's race. The primary evaluation in the emergency room revealed both direct and indirect moderate discomfort of the proximal tibia on the opposite side. The left lower leg did not exhibit any discoloration, edema, or malalignment.

**Figure 2:**
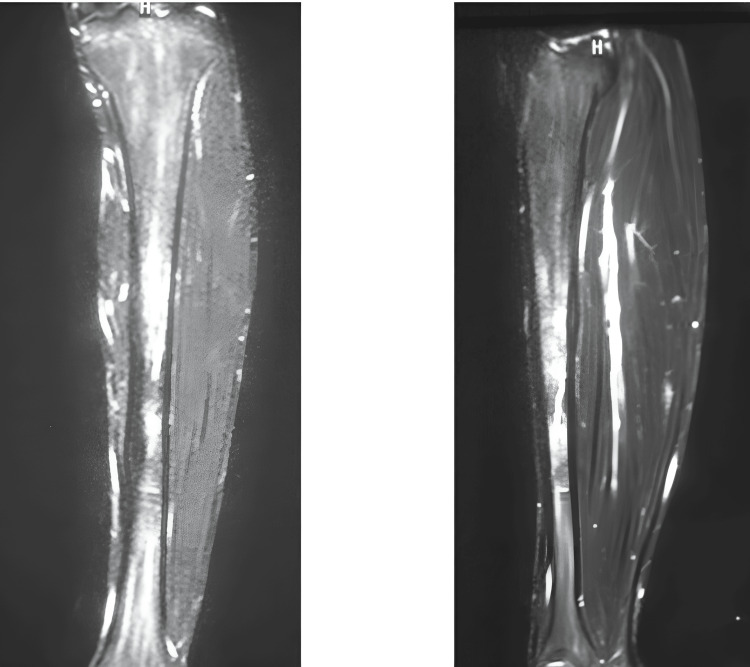
Radiograph of the left lower leg following total fractures of the right leg STIR displays an unusually high signal on imaging. STIR: short tau inversion recovery

A full-thickness fracture within the proximal three-quarters posterior tibial shaft was visible on the imaging. The mid-one-third of the MRI displayed a high-signal region on T1-weighted images, a low signal on T2-weighted imaging, and an unusually elevated signal at the same site on STIR (Figure 2). These results pointed to anomalies including bone marrow hemorrhage and edema. A left tibial stress fracture was identified based on mild buildup shown on bone scintigraphy around the left bottom portion of the thigh.

**Figure 3:**
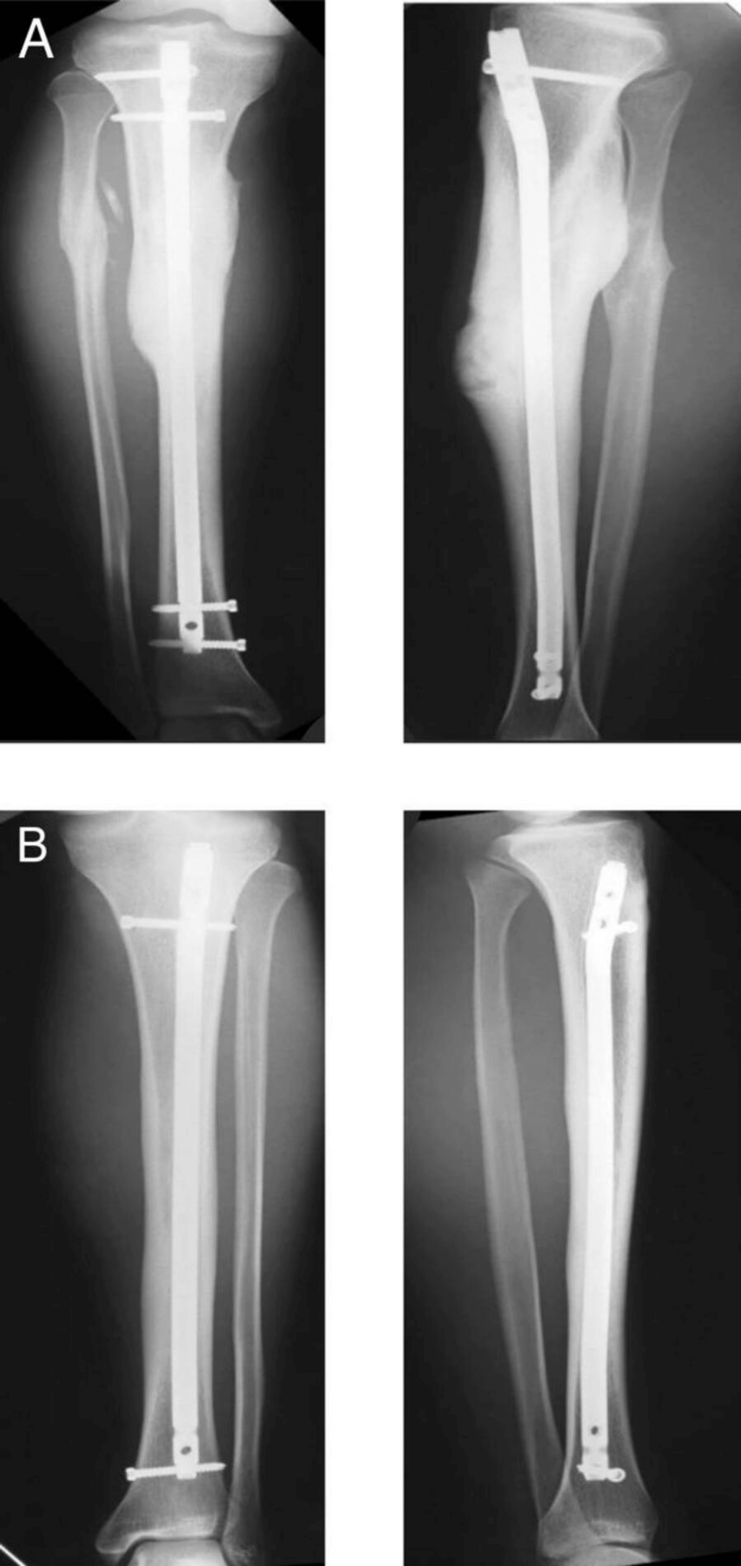
Radiographs demonstrate intramedullary nailing for the total and partial stabilization of tibial fractures

The next day, general anesthesia was used to treat the injuries following written, informed consent from the patient and their parents. In addition, the patient gave written and informed consent for the case, as well as any related photos, to be published. At our institution, there is no ethical approval needed to report specific cases. Intramedullary fixing (10-mm-diameter, T2 Nailing system, Stryker, Kalamazoo, MI, USA) was used in conjunction with closed reduction and internal anchorage of the radial shaft fracture for the left entire tibial fracture and the right tibial stress fracture (Figure 3).

A range-of-motion ankle cast and nine weeks of no weight-bearing on the injured leg were part of the conservative management of the injury. Healing was monitored with a series of imaging studies. The fracture healed completely as a result of this procedure. Ten months after the nailing, the fracture had healed clinically, the patient exhibited no symptoms, and the fracture line was still visible on scans. The patient was cleared to resume moderate exercise three months after the procedure, and five months later, no symptoms remained. He returned to long-distance racing a year later.

## Discussion

The tibia is the most often affected site of stress fractures in individuals of all ages. Two main forms of stress fractures are usually discussed: fatigue fractures, which happen when excessive forces act on bone structure normally and are frequently observed in active individuals like athletes and army recruits. Furthermore, abnormal bone might develop insufficient fractures due to normal forces applied to it. The latter are frequently brought on by systemic illnesses that weaken bone tissue. These include scurvy, past local radiation, osteoporosis, rheumatoid arthritis, hypothyroidism, osteomalacia, type 2 diabetes, fibrous dysplasia of the bones, Paget's disease of the bones, incomplete osteogenesis, and gross proximal fractures of the affected bones. When symptomatic, it is uncommon for inadequacy fractures to only affect the tibia [8].

The patient in this case had posterior tibial fractures, but he kept exercising despite discomfort in his lower legs, resulting in symmetrical tibial stress fractures, one side entire and the other incomplete, which happened at the same time. As far as we are aware, no prior findings of a posterior tibial stress fracture that was as complete as the one in this particular case have been made. Since rest only temporarily alleviates the symptoms, many people might decide not to seek medical attention. Furthermore, the bone may be subjected to extra stresses due to intrinsic components, or internal forces. In addition to unique medical conditions like osteoporosis and low transmit impact forces to the tibia, raising the possibility for a tibial stress injury, these fundamental variables include anatomical modifications, shoes, running mechanics, training plans, and athletic surfaces [2].

The majority of individuals who experience stress fracture symptoms decrease the quantity, frequency, and severity of their daily activities [9]. Stress fractures are more common in long-distance runners due to their high impact and recurrent loads. If progressive bone strain from repeated mechanical stresses of the bone is chronically larger than bone healing, it can lead to stress fractures and bone deterioration [10]. Complementary rest is the usual treatment for posterior tibial stress fractures, which are particularly common among long-distance runners. These fractures are typically treated as low-risk alternatives to anterior fractures and occur due to the compression posterior surface of the distal and distal thirds of the tibia. The first steps in the noninvasive treatment of posterior tibial stress fractures are rest and discontinuation of the exacerbating activity. An osteoblast's inability to keep up with an osteoclast's activity results in a stress fracture, characterized by a mechanical breakdown of the bone [7]. The bone experiences cyclical, repetitive stress with little recovery, making it unable to mend itself in between workouts. Small fractures may form in the bone tissue when a bone is heavily loaded. It is believed that these microcracks help initiate the process of remodeling, which is necessary for the bone to adjust to the operational demands placed on the tissues by overloading. However, excessive loading, in terms of both amplitude and frequency, can result in the development of a stress fracture because remodeling cannot adequately heal the microcracks. However, running's high loading and constant motion make it perfect for stress fractures to happen. Furthermore, significant fractures may lead to a complete fracture [9].

In this particular case, after a year of intermittent rest and competition, a full fracture eventually developed. Earlier return to running may have been achievable by limiting excessive exertion and making sure there is enough rest when lower-body pain develops. Due to the case's gradual onset and modest clinical symptoms, the use of analgesic medicine may have delayed the diagnosis in this case, potentially leading to a complete fracture. To enable a speedy return to training, the intramedullary nailing for the opposing tibial stress fracture was done concurrently.

## Conclusions

Based on our findings, the posterior tibial stress fracture is more prevalent as compared to the anterior stress fracture and is thought to have an excellent likelihood of returning to athletics. However, while complete fractures are uncommon, they can occur in certain cases, and when excessive exercise persists, close monitoring is required. A proximal tibia locking plate was used to fix the bilateral proximal medial epiphyseal tibia stress fractures that were detected on MRI in the case study. Before surgery, the patient stated his desire to resume his active lifestyle after trying conservative measures like rest and compression sleeves without success. The decision to undergo surgery was made because conservative treatments had failed and there were risk factors for poor fracture healing. The long-term effects are currently unknown.
